# Generation of a novel mouse strain with fibroblast-specific expression of Cre recombinase

**DOI:** 10.1016/j.mbplus.2020.100045

**Published:** 2020-07-07

**Authors:** Jahedul Alam, Moses Musiime, Andreas Romaine, Mugdha Sawant, Arne Olav Melleby, Ning Lu, Beate Eckes, Geir Christensen, Donald Gullberg

**Affiliations:** a Department of Biomedicine and Center of Cancer Biomarkers, University of Bergen, Bergen, Norway; b Institute for Experimental Medical Research, Oslo University Hospital and University of Oslo, Oslo, Norway; c KG Jebsen Center for Cardiac Research, University of Oslo, Oslo, Norway; d Center for Heart Failure Research, Oslo University Hospital, Oslo, Norway; e Translational Matrix Biology, University of Cologne Medical Faculty, Cologne, Germany

**Keywords:** Integrin alpha11, Fibroblast, Cre-recombinase, Integrase, Fibroblast-specific

## Abstract

Cell-specific expression of genes offers the possibility to use their promoters to drive expression of Cre-recombinase, thereby allowing for detailed expression analysis using reporter gene systems, cell lineage tracing, conditional gene deletion, and cell ablation. In this context, current data suggest that the integrin α11 subunit has the potential to serve as a fibroblast biomarker in tissue regeneration and pathology, in particular in wound healing and in tissue- and tumor fibrosis. The mesenchyme-restricted expression pattern of integrin α11 thus prompted us to generate a novel ITGA11-driver Cre mouse strain using a ϕC31 integrase-mediated knock-in approach. In this transgenic mouse, the Cre recombinase is driven by regulatory promoter elements within the 3 kb segment of the human *ITGA11* gene. β-Galactosidase staining of embryonic tissues obtained from a transgenic ITGA11-Cre mouse line crossed with *Rosa 26R* reporter mice (ITGA11-Cre;R26R) revealed ITGA11-driven Cre expression and activity in mesenchymal cells in a variety of mesenchymal tissues in a pattern reminiscent of endogenous α11 protein expression in mouse embryos. Interestingly, X-gal staining of mouse embryonic fibroblasts (MEFs) isolated from the ITGA11-Cre;R26R mice indicated heterogeneity in the MEF population. ITGA11-driven Cre activity was shown in approximately 60% of the MEFs, suggesting that the expression of integrin α11 could be exploited for isolation of different fibroblast populations. ITGA11-driven Cre expression was found to be low in adult mouse tissues but was induced in granulation tissue of excisional wounds and in fibrotic hearts following aortic banding. We predict that the ITGA11-Cre transgenic mouse strain described in this report will be a useful tool in matrix research for the deletion of genes in subsets of fibroblasts in the developing mouse and for determining the function of subsets of pro-fibrotic fibroblasts in tissue fibrosis and in different subsets of cancer-associated fibroblasts in the tumor microenvironment.

## Highlights

• A mouse strain with Cre-recombinase driven by the human integrin α11 promoter has been generated.
• Cre-recombinase expression in this strain has been characterized using the Rosa26R reporter mouse.
• ITGA11-Cre is restricted to fibroblast subsets in mouse embryos, skin wounds and fibrotic hearts.
• This Cre-driver strain will be a useful tool to study role fibroblasts in fibrosis and tumors.

## Introduction

Integrin α11β1 is a collagen-binding integrin which has been shown to play a crucial role during myofibroblast differentiation in healing wounds [1,2]. In past years there has been an increased awareness about the similarities between wound healing, fibrosis and tumor-stroma interactions [[3], [4], [5]]. Recent data suggest that α11 in addition to playing a fundamental role in granulation tissue formation and myofibroblast differentiation, is also pro-tumorigenic in non-small cell lung cancer and breast cancer stroma and pro-fibrotic in mouse hearts [[6], [7], [8]].

The expression pattern of α11 in normal and pathological mouse tissues has so far largely been analyzed with polyclonal antibodies and radioactive in situ hybridization techniques [9,10]. The data obtained using these methods demonstrated a mesenchymal expression pattern of integrin α11 confined to a subset of fibroblasts. More recent data indicated expression in a subset of mesenchymal stem cells [11]. Although immunostaining of α11 in normal adult tissues using polyclonal antibodies is problematic due to low expression and background staining, successful immunostaining for α11 was performed on human fetal and mouse embryonic mesenchymal cells using species-specific polyclonal α11 antibodies [9,10]. An expression of α11 in subsets of mouse and human mesenchymal stem cells has also been established [11,12].

Early studies in human tissues indicated restricted expression of α11 in adult tissues but induction in the stroma of non-small cell lung cancer and head and neck squamous carcinoma [13,14]. More recently the generation of monoclonal antibodies (mAbs) directed against human α11 has increased the specificity and therefore accuracy of detecting α11 expression in human cells and tissues [15,16]. Data using the integrin α11 mAbs so far indicate different degrees of expression of integrin α11 in the stroma of breast, ovary, skin, lung, uterus, stomach and pancreatic ductal adenocarcinoma (PDAC) tumors [16].

Based on the distinct expression pattern of integrin α11, we set out to generate an integrin α11 promotor-driven Cre mouse strain (ITGA11-Cre) that could be used both for cell lineage tracing, for α11-specific gene deletion and ITGA11-Cre-directed cell ablation [17]. We have previously determined that *in vitro* 3 kb of the human integrin α11 promoter retains expression in mesenchymal cells and that basal activity is governed by Sp1 and Ets-1 transcription factors, whereas TGF-β responsiveness was demonstrated to depend on a conserved E-box with features of a Smad-binding site [18,19]. Also *in vivo* the crucial elements for mesenchymal cell-specific expression seem to be contained in the 3 kb promoter region, which was confirmed in transgenic reporter mice where lacZ reporter expression driven by 3 kb *ITGA11* promoter was observed in a fibroblast-specific pattern [19]. Based on these data we chose to use the same 3 kb promoter region to generate an ITGA11-Cre driver strain. Integration of the ITGA11-Cre transgene in the mouse genome was accomplished using the ϕC31 integrase method [20]. We herein describe the first characterization of the ITGA11-Cre driver strain and analysis of functional Cre using ROSA 26R reporter mice [21]. In agreement with our previous data obtained with ITGA11-lacZ reporter mice, our current data demonstrate that 3 kb of the *ITGA11* promoter is sufficient to drive functional Cre recombinase expression in a pattern that is indistinguishable from the endogenous α11 expression as detected by immunochemistry. We also demonstrate a heterogeneity in ITGA11-Cre expression in cultured mouse embryonic fibroblasts and demonstrate that the ITGA11-Cre, like the endogenous integrin, is induced in a subset of fibroblasts in wound granulation tissue and in fibrotic heart tissue.

## Results

### Generation of ITGA11-Cre mouse strain using site-specific integrase-mediated transgenesis

**Fig. 1:**
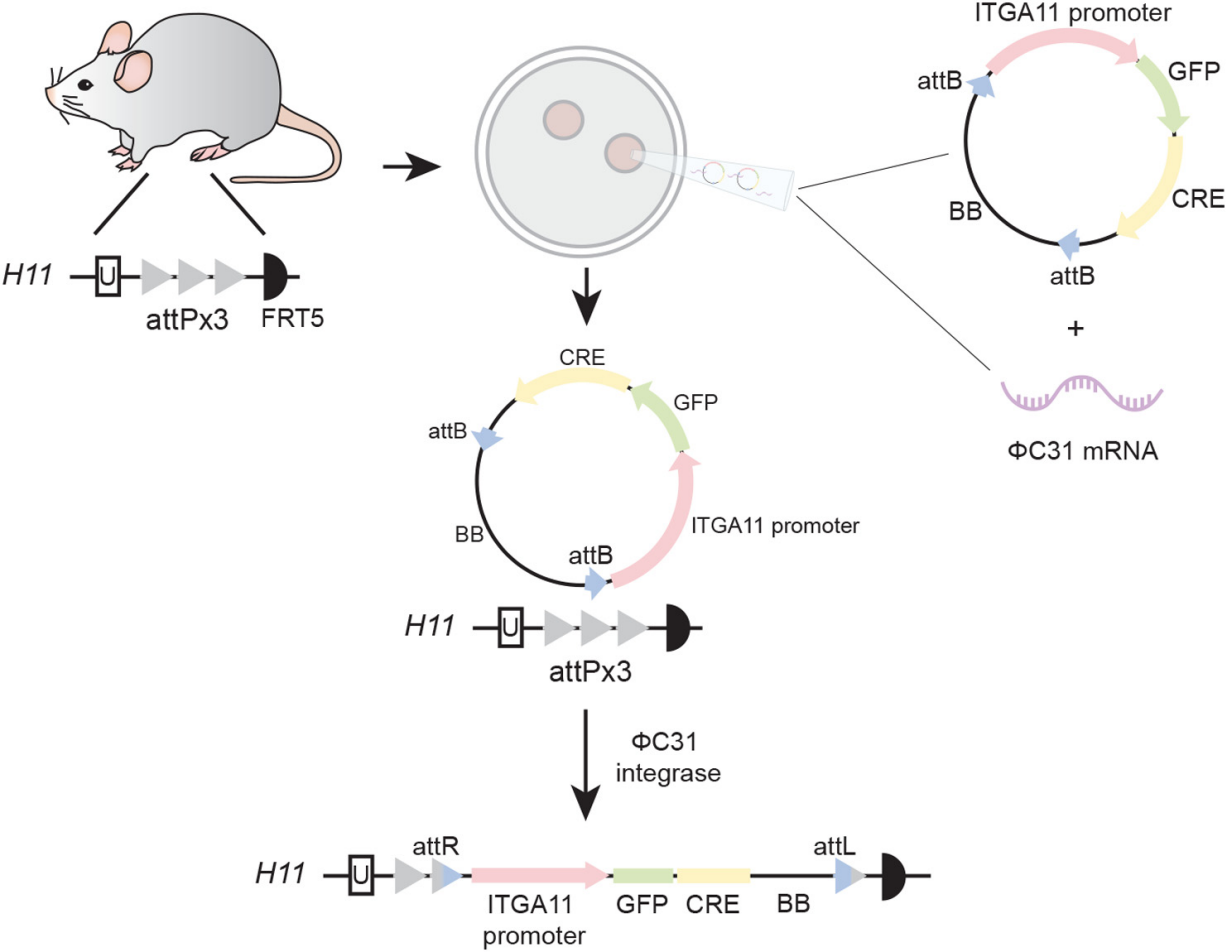
Schematic illustration of site-specific insertion of the ITGA11-Cre transgene into the *H11P3* locus in mouse genome. C57BL/6 mice homozygous for the modified *H11* locus served as embryo donors. The plasmid where the transgene ITGA11-GFPCre is flanked by two attB sites was mixed with in vitro transcribed ϕC31 mRNA and injected into a single pronucleus of each zygote. Insertion of the transgene at the attP site was mediated by ϕC31. Integration of the plasmid bacterial backbone (BB) was not a planned outcome, but according to expression analyses seemed to have little effect on transgene expression.

Our previous work indicated that the 3 kb sequence of the human *ITGA11* promoter reflects the endogenous fibroblast-specific α11 integrin expression pattern. [19]. To generate a novel ITGA11-Cre driver mouse we chose to use the same *ITGA11* promoter region and the ϕC31 integrase knock-in method which ensures single integration of the transgene at a specific *H11P3* gene locus between two attP sites (Fig. 1). PCR-based genotyping indicated a correct integration of the *ITGA11* promoter and sequencing of the *H11* locus also demonstrated integration of the vector backbone (not shown). Previous studies have demonstrated that bacterial backbone can influence expression level, without necessarily impairing stable non-toxic moderate Cre transgene expression [20].

### Characterization of ITGA11-Cre expression pattern

To determine if the integrated *ITGA11-Cre* promoter construct resulted in active Cre recombinase we crossed the ITGA11-Cre mice with the reporter Rosa26R mouse (R26R). In the R26R mouse strain the inserted β-galactosidase (β-gal) sequence in the *Rosa26* locus is disrupted by insertion of a *loxP-*neo*mycin* resistance cassette [21]. The *lacZ* gene can be reactivated through Cre-mediated excision of the *loxP-neo* sequence. When whole mount X-gal staining was performed to monitor β-galactosidase activity in E13.5 embryos, only embryos expressing ITGA11-Cre as judged by genotyping demonstrated a specific blue staining (Supplementary Fig. 1). Closer examination in a stereomicroscope revealed that X-gal staining was largely restricted to the dermis and did not extend to internal organs. We reasoned that this most likely was due to poor accessibility of staining reagent to internal organs, and that tissue sectioning would be needed in order to visualize β-galactosidase activity in internal tissues of ITGA11-Cre mice.

### Cre expression in fibroblasts of the musculoskeletal system

**Fig. 2:**
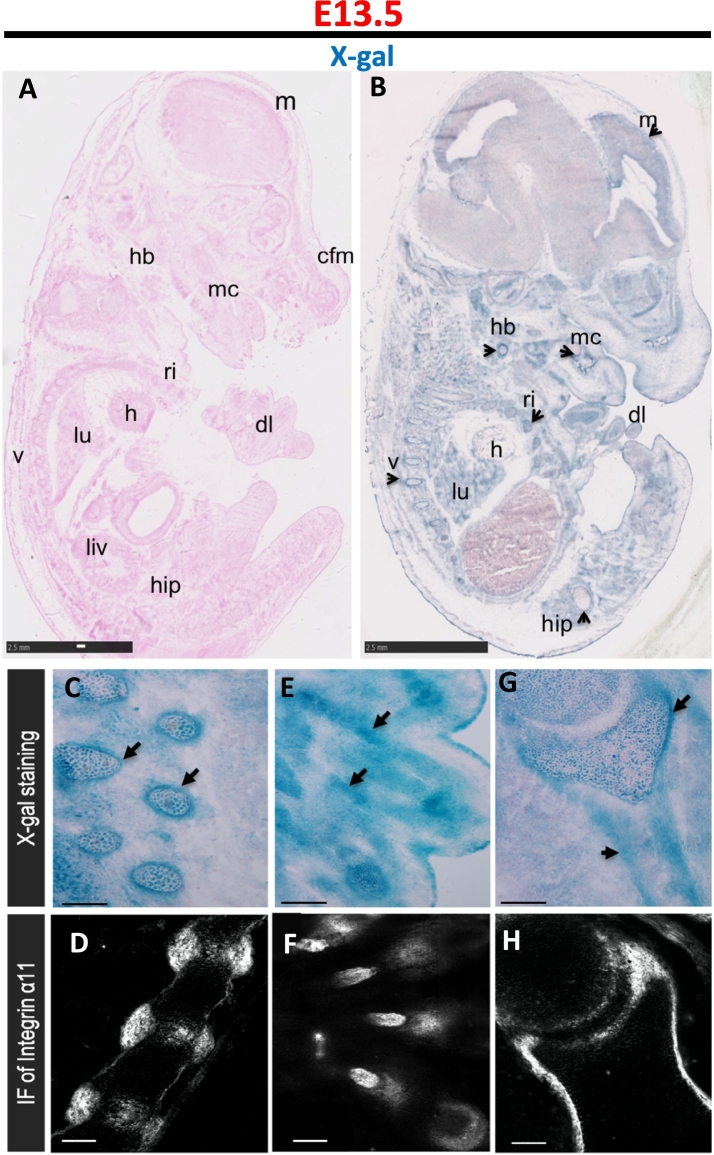
Cre expression indicated by *LacZ* reporter gene expression in E13.5 mouse embryos resemble the endogenous expression pattern of integrin α11. Cryosections of Cre-positive (ITGA11-Cre+;R26R) embryos were analyzed for β-galactosidase activity. Strong X-gal staining was noted in the periosteum and cartilage primordium of vertebrae (v), digit of limb (dl), ribs (ri), hip bone, hyoid bone (hb), and heart (h), lung (lu) and Meckel's cartilage (mc) in agreement with expression of endogenous α11 expression at these sites (B). No X-gal staining was visible in the Cre negative (ITGA11-Cre-;R26R) embryos (A). X-gal staining and immunostaining of integrin α11 were performed on sagittal embryo cryosections (C–H). LacZ and integrin α11 (arrows) are expressed in the mesenchymal condensation/periosteum of vertebrae (v) and intervertebral disc (C, D), mesenchyme in forming digits (E, F), periosteum of scapula (sp) (G, H). Scale bars represent 2.5 mm in A and B, 100 μm in C to H.

**Fig. 3:**
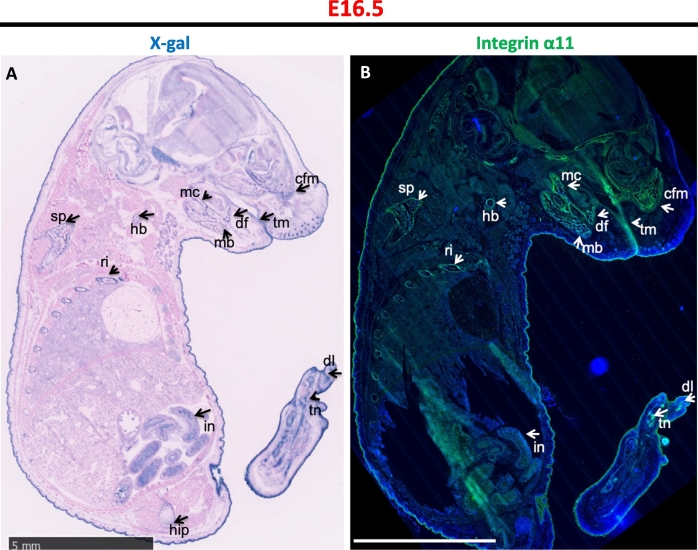
Cre expression in E16.5 mouse embryos. Embryos were harvested from ITGA11-Cre+;R26R and ITGA11-Cre-;R26R mice at E16.5. β-galactosidase activity (A) and endogenous expression of integrin α11 (B) were analyzed in embryo cryosections. X-gal positive staining similar as Fig. 2B was observed in the ITGA11-Cre+;R26R embryo (A), scale bar = 5 mm.

Sagittal sections were prepared from the cryopreserved ITGA11-Cre;R26R embryos at E13.5 and E16.5. Both β-galactosidase activity and endogenous expression of integrin α11 were analyzed in these cryosections. In the E13.5 embryos, Cre activity was detected in sections from the ITGA11-Cre+;R26R mice but not in sections from the ITGA11-Cre-;R26R mice (Fig. 2A, B). Strong X-gal staining was noted in fibroblasts of the skeletal system including the periosteum and cartilage primordium of vertebrae, intervertebral disc and scapula, and in mesenchyme in forming digits. Hence, the observed X-gal staining in transgenic mice is in agreement with previously observed embryonic expression of endogenous α11 at these sites [10,22] (Fig. 2C–H). A similar pattern of Cre activity was observed in E16.5 embryos in fibroblasts in tissues including cartilage primordium of ribs, hyoid bone, hip, scapula, digits of limb, Meckel's cartilage, mandible bone, temporal bone, orbitosphenoid bone, and in dental follicle that later forms the periodontal ligament and tendon, again reflecting previously reported endogenous integrin α11 expression (Fig. 3).

### Cre activity in forming fibroblasts outside the musculoskeletal system

In addition to the observed X-gal staining in mesenchymal cells in the forming skeletal system, X-gal staining was also noted in meningeal fibroblasts covering the brain and, in the epicardium, (Fig. 2). This expression also agrees with that seen by in situ hybridization and immunostaining of mouse embryos with reagents specific for α11 RNA and α11 protein [10]. The blue color seen inside the brain does not agree with endogenous α11 expression and is typically not observed in control embryos, and thus deserves further studies. Endogenous β-galactosidase activity noted in intestine and skin epidermis represents non-specific staining since control Cre negative embryos displayed a comparable X-gal staining pattern.

**Fig. 4:**
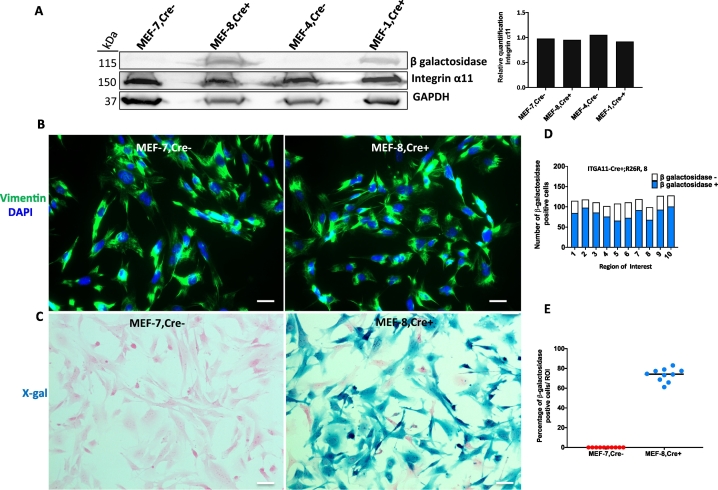
Cre is functional in MEFs isolated from ITGA11-Cre;R26R mouse embryos. Western blot showing the endogenous integrin α11 expression level and the expression of β-galactosidase to indicate the Cre activity in the MEF isolates from E13.5 embryos of the transgenic mouse (A). Representative vimentin staining (B) and X-gal staining (C) of the MEFs isolates from either Cre+ or Cre− embryos. Quantification of the MEFs X-gal staining by cell number (D). Cre+ and Cre− MEFs were counted and quantified from ten staining fields of each MEF isolates (five fields from each of the duplex coverslips). The percentage of X-gal-positive cells in each field was summarized in (E). Note absence of β-galactosidase band (A) or X-gal staining (D) in Cre-negative MEFs. Scale bar = 20 μm.

We have previously reported that integrin α11β1 is a major collagen receptor on mouse embryonic fibroblasts [10,22]. Cre recombinase activity was thus tested in MEFs cultured from our transgenic mice. A robust expression of β-galactosidase was observed in vimentin +- MEFs harvested from ITGA11-Cre+;R26R littermate embryos at 13.5 days [7,23]. Interestingly, Cre activity was not detected in all but in approximately 60% of these vimentin +- MEFs (Fig. 4).

### Low expression of ITGA11-Cre in adult mouse tissues

**Fig. 5:**
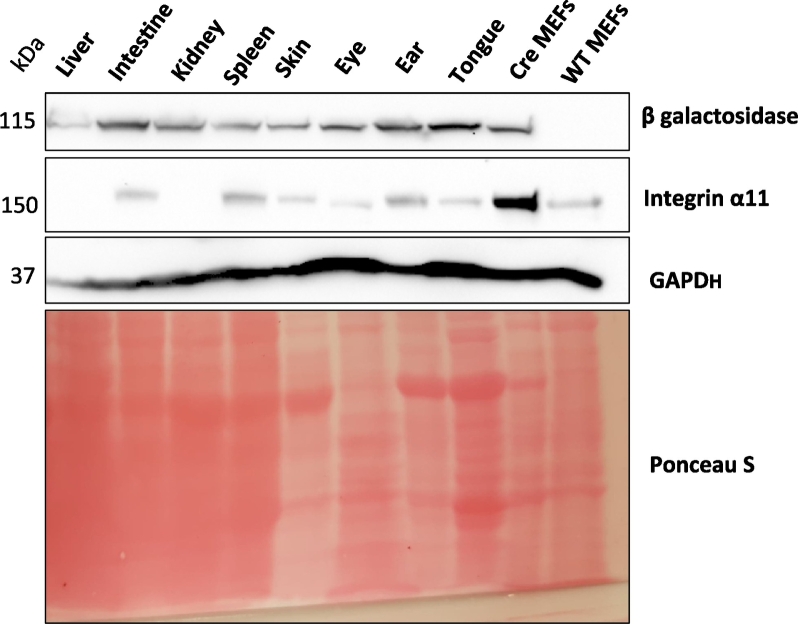
Low expression of ITGA11-Cre in adult mouse tissues. Western blot analysis showing the total protein expression of β galactosidase and integrin α11 in the different adult tissues harvested from ITGA11-Cre+;R26R mice. MEFs from transgenic and wild-type mice, respectively, were used as positive and negative controls. GAPDH and Ponceau S stained total proteins were used as loading controls.

We have previously determined that integrin α11 expression is low in adult tissues [22]. In order to analyze Cre activity in the transgenic adult mouse tissues, organ lysates from adult mice were tested for β-galactosidase by Western blotting. ITGA11-Cre+;R26R mouse adult tissues including intestine, spleen, eye, ear, skin and tongue expressed β-galactosidase, which is in agreement with endogenous integrin α11 expression (Fig. 5). Interestingly, β-galactosidase was detected in kidney and liver, without a corresponding signal for Cre-recombinase. The endogenous nature of the kidney β-galactosidase expression was confirmed in ITGA11-Cre-;R26R kidney sections (Supplementary Fig. 2).

### Induction of ITGA11-Cre activity in fibrotic hearts

**Fig. 6:**
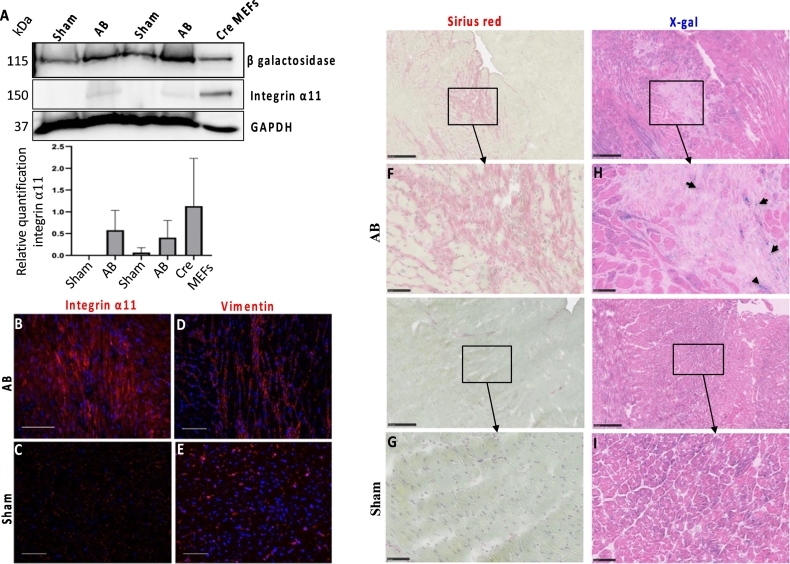
Induction of ITGA11-Cre in fibrotic hearts. Aortic banding was performed on 8 weeks old ITGA11-Cre+;R26R mice in a protocol resulting in fibrosis. Sham-operated mice were used as controls. (A) The total protein expression of β galactosidase and integrin α11 in both sham- and aortic-banded hearts were analyzed by western blotting. Adjacent heart cryosections were stained with Sirius red (F, G) and X-gal (H, I) from fibrotic regions of aortic banded hearts and the ventricular region of sham-operated heart. In addition, adjacent cryosections were immune-stained with integrin α11 (B, C) and vimentin (D, E), respectively from fibrotic regions of aortic banded hearts and the ventricular region of sham-operated heart. Induction of Cre-activity was observed in the fibrotic area of aortic banded hearts compared to sham-operated heart. Scale bar = 100 μm.

Since we established a role of integrin α11β1 in fibrotic skin and heart [7,23], we investigated the *ITGA11* promoter-driven Cre-recombinase activity in fibrotic hearts that developed upon aortic banding (AB) in 8 weeks old ITGA11-Cre+;R26R mice. The AB results in increased left ventricular afterload in the heart, resulting in fibrosis. Sham-operated mice were used as controls. The levels of β-galactosidase and integrin α11 were examined in both sham-operated and aortic banded hearts by western blotting (Fig. 6A). Adjacent heart cryosections from the fibrotic left ventricular regions of AB hearts and the ventricular region of sham-operated hearts were analyzed by X-gal staining. The induction and pathological organization of fibrillar collagens in fibrotic areas was visualized by Sirius red staining. β-Galactosidase activity above background was identified in a few isolated cells in the fibrotic area of AB hearts which agrees with a previous study of α11 expression in the heart [7] (Fig. 6 F–I). Immunostaining and Western blotting confirmed a prominent, but restricted, expression of α11 in fibrotic left ventricular regions of aortic-banded hearts compared to the ventricular region of sham-operated hearts (Fig. 6B–E).

### Induction of ITGA11-Cre activity in the granulation tissue of healing skin wounds

**Fig. 7:**
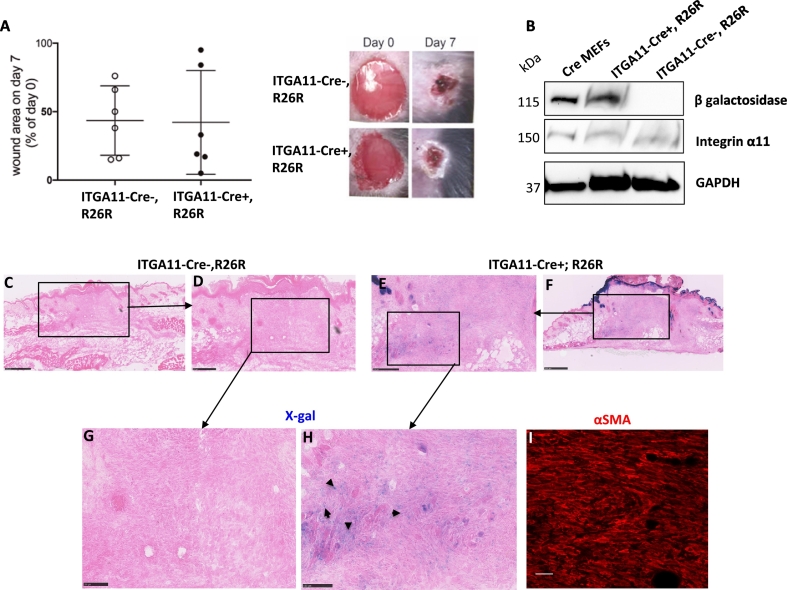
Induction of ITGA11-Cre in the granulation tissue of healing skin wounds. Two circular wounds of 6 mm diameter were inflicted on the backs of ITGA11-Cre+; R26R and ITGA11-Cre-; R26R mice and harvested on day 7 post injury. (A) Macroscopic aspect of wounds taken immediately following wounding (day 0) and of contracted wounds with scab at day 7. (B) Western blot showing protein levels of β-galactosidase and integrin α11 in lysates of day7 wounds. MEF lysates from Cre-positive mice were used as positive control. β-galactosidase activity was detected in ITGA11-Cre+; R26R but not in Cre-negative wounds. (C–F) Low magnification overview of wounds at day7 is shown, boxes indicate areas shown at higher magnification in (G, H). (I) Consecutive wound sections were immunostained with antibodies to αSMA to visualize wound myofibroblasts (I). Scale bars represent 500 μm in (C, F), 250 μm in (D, E) and 100 μm in (G–I).

We have previously determined that integrin α11β1 fulfils an important function in myofibroblast differentiation and function during the repair process of excisional wounds [2]. To examine ITGA11-Cre expression during wound healing, Cre-recombinase activity was assessed in excisional wounds at 7 days after injury, coinciding with abundant appearance of wound myofibroblasts. The course of healing was comparable in ITGA11-Cre+;R26R and ITGA11-Cre-;R26R mice as judged by macroscopic wound closure and scab formation (Fig. 7A). Wounds were harvested at 7 days after wounding without wound margins and lysed for protein analysis. Western blotting confirmed the expression of detectable β-galactosidase only in the Cre-positive wound samples (Fig. 7B). X-gal staining of sections cut from the wound center demonstrated Cre-recombinase activity in the ITGA11-Cre+;R26R, but not in control Cre-negative wounds (Fig. 7C–H). Comparison with αSMA staining of parallel tissue sections indicated that only a subset of αSMA-positive cells demonstrated Cre-recombinase activity (Fig. 7G–I).

## Discussion

In recent years the pivotal role of fibroblasts in wound healing, fibrosis and tumor-stroma interactions has become increasingly recognized [3,[24], [25], [26], [27], [28], [29]]. Although it has been known for some time that fibroblasts in tissues are heterogeneous, the recent use of cell lineage tracing and single cell RNA sequencing has demonstrated a surprisingly high degree of fibroblast heterogeneity, especially in tissues undergoing reorganization following tissue damage or disease [4,[30], [31], [32]]. The lack of cell-specific markers and antibodies reactive with fibroblasts and activated fibroblasts poses a serious challenge when evaluating the role of fibroblasts in these events. Thus proteins such as Thy-1 [33], fibroblast-specific protein-1 (FSP-1) [34], fibroblast activation protein (FAP) [35], EDA-fibronectin [36], transcription factor 4 (Tcf4) [37] α-smooth muscle actin (αSMA) [38], vimentin [39], PDGF receptors [40] and prolyl hydroxylase and collagen I [41] have all been used as fibroblast and/or myofibroblast-specific biomarkers, but are all expressed by other cell types as well. Cre-driver mice based on the regulatory elements of these genes will thus also display recombinase activity in non-fibroblast cells [[42], [43], [44], [45]]. To specificity of Cre-driver strains, selective parts of different promoters have been used to increase selective expression in fibroblasts, exemplified by collagen I, where Cre-driver strains (based on different parts of the *Col1a1* and *Col1a2* promoters) exist with preference for osteoblasts and fibroblasts, respectively [46,47].

In the current study we have used 3 kb of the human *ITGA11* promoter to drive Cre recombinase expression. We have previously characterized this *ITGA11* promoter fragment in transgenic mice, showing that it is able to drive expression of a reporter gene which phenocopies the endogenous embryonic expression of α11 [19]. However, it was not known whether this *ITGA11* promoter fragment would also display increased activity during excisional skin wound healing and aortic banding of hearts, two conditions characterized by elevated integrin α11 protein expression [2,7]. We have previously demonstrated that the *ITGA11* promoter in cultured cells is induced by TGF-β via an E-box positioned upstream of two tandem Sp1 binding sites (approximately 200 nucleotides upstream of the transcription start in the *ITGA11* promoter) [19]. The in vivo data presented herein now suggest that 3 kb of the human *ITGA11* promoter hosts all the elements needed to replicate endogenous expression of α11 both during development and in wound healing and heart fibrosis, situations both characterized by increased TGF-β activity. The ITGA11-Cre strain will enable gene deletion in α11-expressing cells. The ability of a relatively restricted *ITGA11* promoter fragment to drive cell type specific Cre-recombinase activity is thus reminiscent of *Col1a1* and *Col1a2* promoters capable of driving Cre-recombinase expression in osteoblasts and fibroblasts, respectively.

In addition to serving as a tool for cell type specific gene deletion, the ITGA11-Cre driver mice can also be used for cell lineage tracing [48] and ITGA11-Cre directed cell ablation [17]. Cell lineage tracing in mice has both clarified the heterogeneity of fibroblasts in multiple tissues [4] including the skin and heart as well as demonstrated the selective contribution of specific subsets of fibroblasts to wound healing and cardiac fibrosis, respectively. In mouse skin wounds, as much as 30% of the myofibroblast-like cells are estimated to be contributed by activated NG2-positive pericytes [49]. Other studies have recently used cell lineage tracing to identify papillary dermal and reticular dermal fibroblasts with distinct biomarker profiles in mouse [24,26], which appears distinct from corresponding profiles in human dermal fibroblasts.

During the repair process of skin wounds endogenous fibroblasts migrate into and fill up the tissue defect. The immigration of two major fibroblast phenotypes in wounded mouse skin occurs dynamically in two waves [50]. In a careful separate study these fibroblasts were further grouped into a “PDGFRα^high^ subset” and a “PDGFRα^low^ subset”, which could be further subdivided into a total of 12 clusters [51]. As healing is complete, wound fibroblasts are thought to disappear through apoptosis [52]. In mouse skeletal muscle and in skin, ADAM12^+^;PDGFRα^+^ perivascular cells appear to play an important role in tissue repair [53]. Recent data also demonstrate a role of Gli^+^ mesenchymal stem cells (MSCs) in dermal wound healing [54]. Our analysis of ITGA11-Cre activity in skin and skin wounds demonstrates weak staining in adult mouse skin that corresponds with integrin α11 protein distribution, and a limited expression in deeper areas of the granulation tissue. These results suggest that only a subset of wound myofibroblasts has strong *ITGA11* promoter activity or that α11-positive wound myofibroblasts display a strictly regulated spatiotemporal distribution, and presumably also function, during the healing process. Recent analysis of cancer associated fibroblasts revealed no or little overlap of α11 and NG2 expression, suggesting that pericytes are not a major source of α11 expressing myofibroblasts [16]. More recent omic-based studies of aging fibroblast and their role during wound healing show that the ratios of different subpopulations change with aging, and that α11 potentially can be used as a marker for activated fibroblasts in such studies [31]. Further experiments are needed to better characterize the origin and identity of the cells marked by ITGA11-Cre in skin and skin wounds. By contrast, more information has become available on the origin of fibroblasts in the heart. Lineage tracing experiments have shown that resident cardiac fibroblasts are derived from embryonic epicardium and endothelial cells that have undergone epithelial-mesenchymal transition (EMT) or endothelial-mesenchymal transition (endMT), respectively [55]. Upon injury to the heart, activated myofibroblasts (derived from resident cardiac fibroblasts) contribute to reactive fibrosis which occurs in the interstitium as well as replacement fibrosis after myocardial infarction where ECM replaces the lost myocardial cells [56]. For both types of cardiac fibrosis, it is important to identify molecular mechanisms and signatures in the pro-fibrotic subsets of fibroblasts in the fibrotic heart.

Our analysis of ITGA11-Cre in normal and hearts subjected to aortic banding reveal low expression in adult heart, but induction in the fibrotic heart. This is in agreement with reports on endogenous α11 expression in pressure overload-induced cardiac fibrosis [7]. In the challenged heart the interstitial fibroblasts fill up damaged areas and during the repair phase express alpha smooth muscle actin (αSMA) and are contractile. Interestingly, these fibroblasts then lose αSMA expression and become quiescent [57]. Similar to the wound response in skin, Gli^+^ MSCs have been shown to contribute to cardiac fibrosis [54]. The expression of α11 is fairly restricted and further analysis is needed to better identify this subset of cardiac fibroblasts.

The finding that ITGA11-Cre is expressed in about 60% of MEFs is in agreement with unpublished observations that α11 is not homogeneously expressed in all MEFs in vitro (data not shown Erusappan and Gullberg, 2019). We have previously observed that mesenchymal cells not normally expressing α11 start to express α11 when placed in culture [3,58], indicating that a strict transcriptional control preventing α11 expression must exist in 40% of the MEFs. This finding also raises the possibility that different fibroblast subsets can be separated based on the collagen-binding integrin repertoire. It will be interesting to determine the integrin repertoire of the ITGA11-Cre/Rosa26R fibroblasts and to identify possible functional differences with regard to pro-fibrotic properties versus fibroblasts in which ITG11-Cre is not active, and which presumably also lack α11 protein expression.

The finding that ITGA11-Cre expression mimics endogenous expression also during wound healing and cardiac fibrosis supports the notion that a majority of regulatory sequences of *ITGA11* are concentrated in the 3 kb region.

Out data suggest that the ITGA11-Cre driver strain will be a useful tool to further sort out the importance of fibroblast subsets in dynamic tissue reorganization events involving fibrosis. A powerful tool to delete cells in a restricted manner involves the tissue specific Cre induction of diphtheria toxin receptor (DTR) and use of diphtheria toxin for cell ablation [59]. Based on our current understanding of integrin α11 we postulate that the α11 expression and function is prominent in pro-fibrotic fibroblasts. To confirm this hypothesis it would be interesting to cross the ROSA26iDTR mice [17] with the ITGA11-Cre mice, which would enable cell ablation in an α11-specific manner.

## Experimental procedures

### Ethics

The mouse experiments conducted in this study were approved by the Norwegian Committee for Animal Research (FOTS project number: 20146151) and were performed in agreement with the Guide for the Care and Use of Laboratory Animals (NIH publication No. 85-23, revised 2011, US). The reporting of experimental procedures and results was performed in accordance with the ARRIVE guidelines [60].

### Mouse breeding and maintenance

Mice used for generating the ITGA11-Cre strain (C57BL/6 with attP knock-in at *H11* loci, *H11P3*; homozygous) were bred and maintained at Stanford University in accordance with their Administrative Panel on Laboratory Animal Care, and the Protocol and the Institutional Guidelines by the Veterinary Service Center at Stanford University. Resulting transgenic mice with ITGA11-Cre inserted at the attP site of a *H11* locus (B6;Tg ITGA11-Cre, heterozygotes) were imported to the animal facility of Bergen University. To characterize the Cre activity and expression pattern driven by the *ITGA11* promoter, the ITGA11-Cre mice were bred with the Gt(ROSA)26 lacZ reporter mice (R26R) mice. The R26R mice were kindly provided by Prof. Clifford Kentros and were transported to our facility from NTNU, Norway (originally from The Jackson Laboratory, Stock No: 003474).

### Construction of the ITGA11-Cre transgene

The 3 kb human integrin α11 (*ITGA11*) promoter region, spanning 2962 bp upstream and 25 bp downstream of the transcription start site (−2962 to +25) faithfully recapitulates the endogenous α11 expression pattern in transgenic lacZ reporter mouse embryos [19]. For construction of the ITGA11-Cre transgene, the same 3 kb promoter region was used and came from the pGL3 (−2987) construct [18]. The coding sequence of Cre came from the pBS594 construct (Addgene, Cat#11956) from which Cre is expressed as a protein fused N-terminally with GFP. The 1.8 kb-GFP-Cre fragment was released from the pBS594 construct by *Mlu*I (blunted) and *Bgl*II and subcloned into the *Sgr*AI (blunted) and BglII site of the pGL3b (−2987) construct to obtain the ITGA11-Cre transgene. To be able to use the site-specific integrase-mediated transgenesis method, we further subcloned the ITGA11-Cre transgene (4.8 kb, released by *Sac*I and *Xba*I, end-blunted) into the pBT378 plasmid (provided by the Transgenic Facility, Stanford University) that was cut with *Hin*dIII and *Eco*RI, end blunted. The final construct for transgenesis (pBT378-ITGA11-Cre) was verified by both endonuclease digestion and sequencing.

### Generation of the ITGA11-Cre transgenic mice

The ITGA11-Cre transgenic mice were generated by the site-specific integrase-mediated transgenesis method [20]. Briefly, super-ovulated homozygous attP-containing C57BL/6 females were bred with corresponding males to generate homozygous attP-containing zygotes. A transgene DNA/ϕC31mRNA mix was microinjected into a single pronucleus and cytoplasm of each zygote by using a continuous flow injection mode. The surviving zygotes were implanted into oviducts of pseudo-pregnant CD1 (Charles River) recipient mothers. One ITGA11-Cre transgene positive founder mouse was identified by genotyping using genomic DNA extracted from tail/ear samples.

### Genotyping of the ITGA11-Cre mice

To identify site-specific and random insertions in F0 animals, three PCRs were performed: one for the 5′-end junction (PCR1), one for the 3′-end junction (PCR2), and one internal to the transgene (PCR3). To further test whether parts of the vector backbone were also inserted, a fourth primer pair was used (PCR4). The primer pairs used for the four PCRs were as follows. For PCR1, forward primer BT425(5′F): GGTGATAGGTGGCAAGTGGTATTC; reverse primer BT436(5′R): ATCAACTACCGCCACCTCGAC. For PCR2, forward primer BT522(3′F): CGATGTAGGTCACGGTCTCG; reverse primer BT387(3′R): GTGGGACTGCTTTTTCCAGA. For PCR3, forward primer H11-2F: CCTTCAGCTGCCCACTCTAC; reverse primer a11-2R: CTATTTCTATGGATTTGCCTATTC. For PCR4, forward primer PR21 5′F: CTGCAAGGCGATTAAGTTGG; reverse primer BT387 3′R: GTGGGACTGCTTTTTCCAGA.

### Aortic banding

ITGA11-Cre;R26R mice were subjected to aortic banding (AB) or sham operation as described [61] (N = 6 per group). Mouse heart sections were obtained from mice subjected to constriction of the ascending aorta (AB mice) or sham operated mice. AB mice were subjected to constriction using 0.61 mm-inner diameter O-rings. SH mice were operated under the same conditions, but no O-ring was inserted around the aorta. Mice were sacrificed 13 days after surgery.

### Skin wounding

Two full-thickness excisional wounds of 6 mm diameter, comprising the epidermis, dermis and subcutaneous adipose tissue including the panniculus carnosus muscle, were created on the shaved backs of 13 weeks old female ITGA11-Cre+;R26R and ITGA11-Cre-;R26R mice as described [2,62] (N = 3 per group). Wounds were left uncovered and harvested at 7 days after injury and bisected cranial-caudally. One half of each wound was processed unfixed for cryosectioning, the other half was fixed for 2 h in 4% paraformaldehyde and processed for paraffin embedding according to standard procedures. Sections were cut from the wound center. Experiments were approved by German veterinary authorities (LANUV NRW, permit 84-02.04.2013.A069).

### Isolation of mouse embryonic fibroblasts

Mouse embryonic fibroblasts (MEFs) were isolated from wildtype and ITGA11-Cre;R26R mouse embryos at E13.5 as described [63]. Isolated cells were cultured at 37 °C and 5% CO_2_ in Dulbecco's modified Eagle medium (DMEM; Gibco®, Thermo Fisher Scientific) with 10% fetal bovine serum (FBS; Gibco®, Thermo Fisher Scientific) and 1% antibiotic-antimycotic solution (Gibco®, Thermo Fisher Scientific).

### X-gal staining of cells to visualize Cre activity

MEFs were seeded onto coverslips for 12 h and fixed with 0.2% glutaraldehyde at room temperature for 10 min. After washing with PBS, cells were incubated with X-gal staining solution (5 mM potassium ferrocyanide, 5 mM potassium ferricyanide, and 1 mg/ml X-gal) at 37 °C for 12 h and cells were counterstained with eosin. Experiments were performed with cells up to passage 3.

### X-gal whole mount embryo staining and staining on tissue sections

X-gal whole mount staining of embryo was performed as described previously [19]. Briefly, embryos were dissected at E14 and fixed in 4% paraformaldehyde (PFA) at room temperature (RT) for 60 min, followed by washing with PBS/0.02% NP-40 for 3 × 10 min at RT. Embryos were then incubated at 37 °C overnight in X-gal staining solution (2 mM MgCl_2_, 0.01% sodium deoxycholate, 0.02% NP-40, 0.1% X-Gal, 5 mM potassium ferricyanide and 5 mM potassium ferrocyanide in PBS). After staining, embryos were washed with PBS for 3 × 15 min at RT with agitation, followed by post-fixation with 4% PFA at 4 °C overnight. Embryos were stored in 4% PFA at 4 °C until further use.

For X-gal staining of tissue sections, cryosections were prepared at a thickness of 10 μm from embryos at E13.5 or E16.5, from adult heart or skin and preserved in OCT compound. The sections were treated with fixation solution (0.2% glutaraldehyde, 5 mM EGTA, and 2 mM MgCl_2_) at 4 °C for 30 min. After washing with PBS containing 2 mM MgCl_2_, 0.01% sodium deoxycholate, and 0.02% NP-40, the sections were incubated with X-gal staining solution (5 mM potassium ferrocyanide, 5 mM potassium ferricyanide, and 1 mg/ml X-gal) at 37 °C overnight. X-gal stained sections were counterstained with eosin. Sections were scanned with slide scanner (Olympus VS120 S6). After acquisition, the images were adjusted and analyzed by using NiS Elements software (Nikon).

### Immunofluorescent (IF) staining

IF staining was performed on either the cryosections of the embryos or cultured MEF cells on coverslips. The sections stained were the consecutive embryonic tissue cryosections as prepared for X-gal staining. For staining, the sections were fixed with methanol for 8 min at −20 °C, followed by rehydration with PBS for 3 × 10 min. Sections were then blocked with 10% goat serum in PBS at RT for 1 h and subsequently incubated overnight with rabbit anti-mouse integrin α11 antibody [10]; (dilution 1:200) or mouse anti- αSMA (Sigma, Cat# A2547, 1:400) or rabbit anti-vimentin antibody (Cell Signalling Technology, Cat# D21H3, 1:200) at 4 °C. After washing with PBS/0.05%Tween 3 × 5 min, sections were incubated with secondary antibody goat anti-mouse IgG Alexa Fluor® 488 (1:500, Abcam, Cat# ab150077) or goat anti-rabbit IgG Alexa Fluor® 594 (Abcam, ab150080, 1:600) at RT for 1 h. For staining of the MEF cells, they were fixed by 4% PFA, washed with PBS for 3 × 10 min and blocked with 5% goat serum in PBS/0.3%Triton-X100 at RT for 1 h. Cells were then incubated with primary antibody rabbit anti-vimentin antibody (Cell Signalling Technology, Cat# D21H3, 1:200) and secondary antibody goat anti-rabbit IgG Alexa Fluor® 488 (Abcam, ab150077, 1:500) using the same procedure as that for the staining of the sections. Afterwards, the sections or the cells were washed with PBS/0.05% Tween 3 × 10 min, mounted with ProLong® Gold mounting media (ThermoFisher, Cat# P36931). The stained sections or cells were studied under a Zeiss Axioscope fluorescence microscope and micrographs were acquired using a digital AxioCam mRM camera (Zeiss).

**Sirius red staining.**

Tissue sections were dewaxed in xylene, rehydrated and transferred to Sirius Red staining solution (Polysciences, cat# 24901) for 75 min at RT. Subsequently, sections were rinsed in 0.01 M HCl dehydrated and cleared in xylene for 2 min each. Sections were counterstained with hematoxylin and mounted with Entellan. Images were processed and analyzed as described above for X-gal staining.

### Western blotting

Cultured MEFs were lysed in SDS-sample buffer (Bio-Rad, Cat# 1610791) with 3% of 2-β-mercaptoethanol (Sigma-Aldrich, Cat# M7154). Tissue lysates were prepared as described previously [7]. After tissue lysis, 2× SDS sample buffer with 3% of 2-β-mercaptoethanol was added to lysates. Both cell and tissue lysates were sonicated using a Vibra-CellTM ultrasonic processor (Sonics and Materials, USA). The lysates were subjected to (7.5% acrylamide) SDS-PAGE electrophoresis after boiling for 5 min at 95 °C, and the proteins were transferred to PVDF membranes using the iBlot® system (BioRad). 5% non-fat dry milk (Marvel, UK) was used to block the membranes. Membranes were incubated with sheep anti-mouse α11 antibody (R&D Systems, cat# AF6498, 1:400) or chicken anti-mouse β-galactosidase antibody (Abcam, Cat# ab9361, 1:800) or GAPDH (Santa Cruz Biotechnology, cat# sc-32,233, 1:1000) and anti-β-actin (Sigma-Aldrich, Cat# A5441) overnight at 4 °C before HRP-conjugated goat anti-mouse or donkey anti-chicken or goat anti-sheep secondary antibodies were added. The membranes were developed using the ECL western blotting systems kit (GE Healthcare) and photographed using the ChemiDoc XRS device and the Quantity One 1-D Analysis Software (Bio-Rad).

The following are the supplementary data related to this article.

Supplementary Fig. 1 Cre activities shown by X-gal whole-mount staining of mouse embryos correlate with genotypes of the embryos. (A) Schematic diagram showing the breeding strategy. ITGA11-Cre heterozygotes were bred with R26R (Rosa 26) reporter mice. Embryos were taken at E13.5 for X-gal whole mount staining to determine Cre-recombinase activity. (B) Photograph shows representative staining results for 9 embryos dissected from one female. (C) Photograph of gel shows the corresponding genotyping results of the 9 embryos. The PCR band in lanes E2-E5, E7 and E8 indicates presence of the transgene (TG) whereas absence of the band indicates the un-recombined wild type allele in lanes E1, E6, and E9. Supplementary Fig. 1

Supplementary Fig. 2 Absence of β-galactosidase expression in adult Cre-negative mouse tissues.
Western blot analysis showing the total protein level of β galactosidase in various adult mouse organs from ITGA11-Cre-;R26R mice. Lysate of MEFs harvested from a transgenic mouse was used as positive control. GAPDH and Ponceau S staining were used as loading controls. Supplementary Fig. 2

## Conflict of interest

The contributing authors declare no conflict of interest.
