# Supplementary figures and images for: Generation of a novel mouse strain with fibroblast-specific expression of Cre recombinase
Source: Matrix Biol Plus. 2020 Jul 7;8:100045. doi: 10.1016/j.mbplus.2020.100045 (PMC7852330; doi:10.1016/j.mbplus.2020.100045)

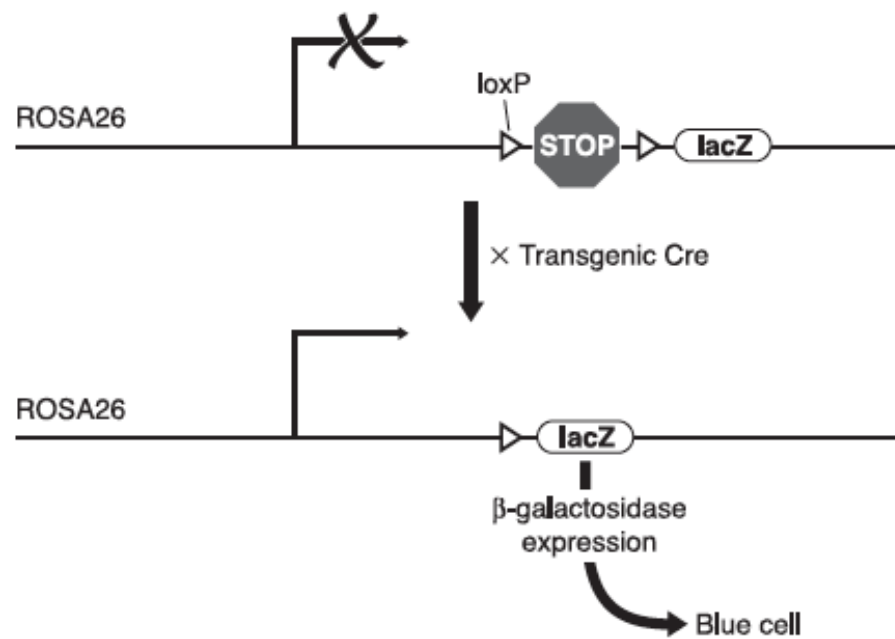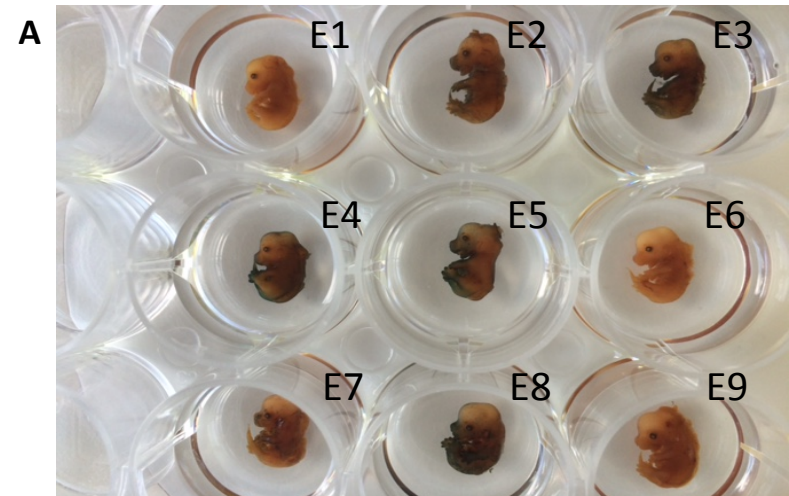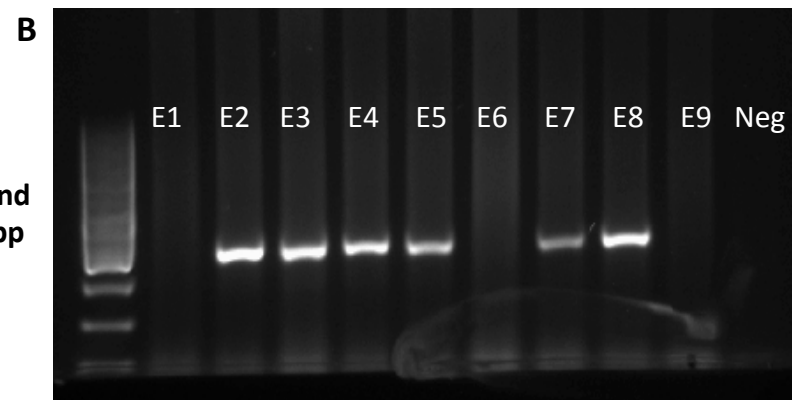

Supplementary figure 1

Supplement: Supplementary Fig. 1 — Cre activities shown by X-gal whole-mount staining of mouse embryos correlate with genotypes of the embryos. (A) Schematic diagram showing the breeding strategy. ITGA11-Cre heterozygotes were bred with R26R (Rosa 26) reporter mice. Embryos were taken at E13.5 for X-gal whole mount staining to determine Cre-recombinase activity. (B) Photograph shows representative staining results for 9 embryos dissected from one female. (C) Photograph of gel shows the corresponding genotyping results of the 9 embryos. The PCR band in lanes E2-E5, E7 and E8 indicates presence of the transgene (TG) whereas absence of the band indicates the un-recombined wild type allele in lanes E1, E6, and E9. [file mmc1.pdf]

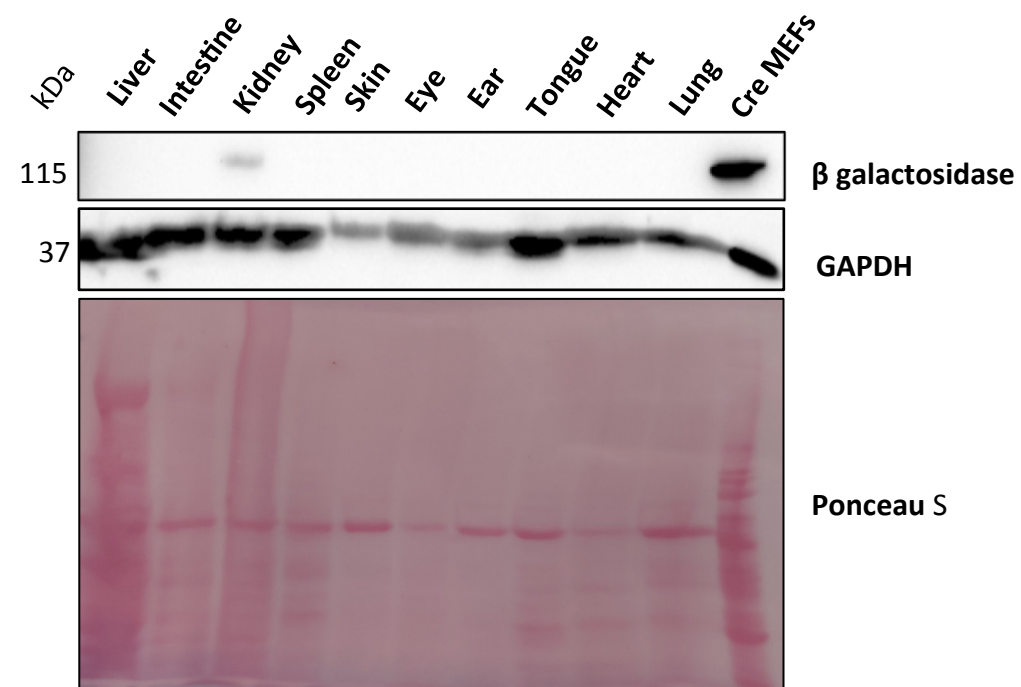

Supplementary figure 2

Supplement: Supplementary Fig. 2 — Absence of β-galactosidase expression in adult Cre-negative mouse tissues. Western blot analysis showing the total protein level of β galactosidase in various adult mouse organs from ITGA11-Cre-;R26R mice. Lysate of MEFs harvested from a transgenic mouse was used as positive control. GAPDH and Ponceau S staining were used as loading controls. [file mmc2.pdf]
